# Procurement and Evaluation of Hepatocytes for Transplantation From Neonatal Donors After Circulatory Death

**DOI:** 10.1177/09636897211069900

**Published:** 2022-01-30

**Authors:** Emil Bluhme, Ewa Henckel, Roberto Gramignoli, Therese Kjellin, Christina Hammarstedt, Greg Nowak, Ahmad Karadagi, Helene Johansson, Öystein Jynge, Maria Söderström, Björn Fischler, Stephen Strom, Ewa Ellis, Boubou Hallberg, Carl Jorns

**Affiliations:** 1 Department of Clinical Science, Intervention and Technology, CLINTEC, Karolinska Institutet, Stockholm, Sweden; 2 Department of Transplantation, Karolinska University Hospital, Stockholm, Sweden; 3 Department of Neonatology, Astrid Lindgren Children’s Hospital, Karolinska University Hospital, Stockholm, Sweden; 4 Division of Pathology, Department of Laboratory Medicine, Karolinska Institutet, Karolinska University Hospital Huddinge, Stockholm, Sweden; 5 Organisation for Organ Donation in Central Sweden, Stockholm, Sweden; 6 Department of Pediatric Gastroenterology, Hepatology and Nutrition, Astrid Lindgren Children’s Hospital, Karolinska University Hospital, Stockholm, Sweden

**Keywords:** neonatal organ donation, warm ischemia time, FRGN mice

## Abstract

Hepatocyte transplantation is a promising treatment for liver failure and inborn metabolic liver diseases, but progress has been hampered by a scarcity of available organs. Here, hepatocytes isolated from livers procured for a neonatal hepatocyte donation program within a research setting were assessed for metabolic function and suitability for transplantation. Organ donation was considered for infants who died in neonatal intensive care in the Stockholm region during 2015–2021. Inclusion was assessed when a decision to discontinue life-sustaining treatment had been made and hepatectomy performed after declaration of death. Hepatocyte isolation was performed by three-step collagenase perfusion. Hepatocyte viability, yield, and function were assessed using fresh and cryopreserved cells. Engraftment and maturation of cryopreserved neonatal hepatocytes were assessed by transplantation into an immunodeficient mouse model and analysis of the gene expression of phase I, phase II, and liver-specific enzymes and proteins. Twelve livers were procured. Median warm ischemia time (WIT) was 190 [interquartile range (IQR): 80–210] minutes. Median viability was 86% (IQR: 71%–91%). Median yield was 6.9 (IQR: 3.4–12.8) x10^6^ viable hepatocytes/g. Transplantation into immunodeficient mice resulted in good engraftment and maturation of hepatocyte-specific proteins and enzymes. A neonatal organ donation program including preterm born infants was found to be feasible. Hepatocytes isolated from neonatal donors had good viability, function, and engraftment despite prolonged WIT. Therefore, neonatal livers should be considered as a donor source for clinical hepatocyte transplantation, even in cases with extended WIT.

## Introduction

Hepatocyte transplantation is an evolving, on-demand treatment for patients suffering from monogenic metabolic diseases, such as deficiency in alpha-1 antitrypsin (A1AT), ornithine transcarbamylase (OTC), or carbamoyl phosphate synthase 1 (CPS-1), or Crigler-Najjar syndrome^1^. These patients are missing a specific enzyme or protein, leading to severe, often fatal, outcomes. However, the liver is otherwise healthy and fully functional. Instead of replacing the whole liver, transplanted hepatocytes can engraft and produce the missing enzyme or protein, reducing or eliminating the underlying disease. In addition, cryopreserved hepatocytes are being considered for treatment of acute liver failure^2^.

Progress in hepatocyte transplantation has been hampered by access to good-quality donor tissue, as most donor organs are used for solid organ transplantation. The majority of livers currently used for hepatocyte isolation are from extended criteria donors—older donors with long warm ischemia time (WIT) and high levels of steatosis, factors that negatively correlate with hepatocyte viability and yield^3–5^.

Previous reports suggest that hepatocytes isolated from neonatal donors may be of superior quality compared to adult hepatocytes. Viability, yield per gram, and function have been reported to be higher in neonatal hepatocytes than adult hepatocytes^6,7^. However, there are concerns regarding maturity^6^. Successful clinical hepatocyte transplantation using neonatal hepatocytes has been reported in four patients with urea cycle disorders^8^. Neonatal hepatocyte transplantation has also been used as a bridge to orthotopic liver transplantation^7^, but previous studies involving neonatal hepatocytes have had limitations on WIT of 30–90 minutes^6,7^.

Neonatal organ donation has not previously been practiced in Sweden for two reasons. First, although a declaration of death according to neurological criteria (DNC) is possible in neonates^9–11^, and Swedish guidelines do not explicitly exclude neonates from DNC guidelines^12^, DNC is currently not applied in neonatal patients in Sweden. Second, donation after circulatory death (DCD) has not been implemented until recently. Thus, neonatal patients have not been considered for organ donation.

In the present study, the aim was to set-up a neonatal donation after circulatory death program in the Stockholm region to procure livers for hepatocyte isolation. We investigated the viability and quality of neonatal hepatocytes and assessed the impact of WIT on viability. We also sought to determine if the cells could be transplanted successfully into suitable recipients and assessed the hepatic gene expression and maturation following transplantation.

## Experimental Procedures

### Donation Process

A research study protocol for neonatal donation after circulatory death was established for the neonatal intensive care units in the Stockholm area. Organ donation was considered in children who died during neonatal intensive care during 2015–2021. With the exception of heart valves, other organs were not considered from donors included in this study.

Exclusion criteria were known maternal infection with viral hepatitis or human immunodeficiency virus. We did not exclude potential donors due to maturation (i.e., preterm birth), birth weight, or long WIT. Inclusion criteria were critically ill neonatal patients in whom further treatment was deemed unethical and futile, and a decision had been made to not initiate or proceed with life-sustaining treatment. The decision to not initiate or continue with life-sustaining treatment was made in accordance with clinical routine after prudent ethical and clinical consideration. This process was carried out prior to and separate from the decision regarding organ donation and inclusion in the study. It was the attending physician’s initiative to ask parents of eligible patients about participation, after which research staff contacted them to provide specific written and oral information about the study. All parents of children participating in this study provided written consent.

Discontinuation of life-sustaining treatment occurred in the neonatal intensive care unit. Following discontinuation, regular auscultations were made until death was determined per clinical routine according to the regulations of the Swedish National Board of Health and Welfare^12^. Death was declared following a clinical examination by a physician auscultating and palpating for an absent pulse, determining no spontaneous breath, and the presence of fixed or dilated pupils. No further treatment or procedures to preserve organ viability were administered before determination of death. Following cardiac arrest, the procurement team awaited the parents’ decision to allow for the surgical procedure to start. A minimum no-touch period of 20 minutes was applied. The liver was procured, as well as cardiac valves from two donors, in accordance with national guidelines for clinical use. The parents were provided the option of having the infant returned to them after the donation procedure.

Time points were recorded for withdrawal of life-sustaining treatment, determination of death, and start of portal perfusion. Agonal time was defined as the time from withdrawal of life-sustaining treatment until declaration of death. WIT was defined as the time from the withdrawal of life-sustaining treatment until start of cold perfusion. In cases in which no withdrawal of life-sustaining treatment was initiated (i.e., in patients with a prenatal diagnosis not compatible with life), WIT started from time of birth. Cold ischemia time (CIT) was defined as the time from cold perfusion until start of digestion of the liver. Total ischemic time was CIT + WIT, from the withdrawal of life-sustaining treatment until commencement of digestion during hepatocyte isolation.

The liver was excised, and the portal vein flushed with histidine-tryptophan-ketoglutarate (HTK; Custodiol^®^, Essential Pharmaceuticals LLC, North Carolina, U.S.) solution. The liver was placed in a sterile bag, submerged in HTK solution, and surrounded by wet ice.

### Clinical Data

Perinatal information and neonatal medical history were retrieved from medical records. Perinatal data included mode of delivery, gestational age at birth, sex, known prenatal diagnosis, and maternal viral hepatitis, human immunodeficiency virus, and syphilis status. Neonatal morbidity included day of life (age when death occurred), inotrope requirement, last known weight, and lab values for alanine aminotransferase (ALT), international normalized ratio (INR), and bilirubin obtained within 1 day prior to donation. Exceptions were biochemical values for donor A and donor K, which were obtained 5 and 3 days prior to donation respectively.

### Hepatocyte Isolation and Storage

A piece of liver was excised and snap-frozen in liquid nitrogen for RNA isolation. Hepatocyte isolation was performed by three-step collagenase perfusion as described previously^2,13,14^, with modification for adaptation to neonatal livers.

Due to the size the portal vein was cannulated rather than utilizing individual liver veins. The liver was placed in a sterile bag in a 37°C water bath, the liver is first perfused with HBSS containing EGTA, after which a second perfusion with HBSS is done to flush out the EGTA. Sequentially a third perfusion containing collagenase is done until the liver shows adequate digestion, with capsule deterioration (9–20 minutes). The tissue is then cut up and diluted with after which the mixture is filtered. Thereafter vials are centrifuged at 1,500 rpm at 5°C which is repeated three times.

Cryogenic preservation and successive thawing were performed according to a previously published protocol^15^.

### Hepatocyte Evaluation

#### Viability and yield

Hepatocyte viability post-isolation and post-cryopreservation was assessed using the trypan blue (Sigma-Aldrich, Missouri, U.S.) exclusion method^13^. Hepatocyte yield was determined immediately after isolation and assessed as million viable cells per gram of liver tissue.

#### Metabolic function

Metabolic activity was measured in fresh and cryopreserved hepatocytes. Phase I metabolism was measured using commercially available luciferin assays, P450-Glo™ (Promega Corporation, Wisconsin, U.S.), for cytochrome P450 1A1, 1A2, 2B6, 2C9, 3A4, and 3A7 as described previously^16^. Luminescence was read directly in a multi-well plate luminometer (CLARIOstar, BMG Labtech, North Carolina, U.S.) and expressed as luminescent counting units (LCU)/min normalized to a million viable cells. Phase II activity was determined by the metabolism of the fluorescent compound resorufin according to a published protocol^13^. Resorufin fluorescent signals were quantified by a spectrophotometer (CLARIOstar BMG Labtech, North Carolina, U.S. excitation: 535 ± 25, emission: 581 ± 20). Conjugation efficiency was quantified by measuring the decrease in the fluorescent signal and reported as the percentage of resorufin metabolized.

#### Apoptosis evaluation

Caspase-3/7 activity was measured by a luminescent assay, Caspase-Glo^®^ 3/7 (Promega Corporation, Wisconsin, U.S.), according to a previously published protocol^16^. Luminescence was read in a Synergy HT luminometer (BioTek Instruments, Vermont, U.S.). Luminescence produced by the assay was proportional to the amount of caspase activity present in the cell sample. Results were expressed as LCU/min and normalized to a million viable cells.

#### Bile acid synthesis

After isolation, hepatocytes were cultured on Matrigel (Sigma-Aldrich, Missouri, U.S.) with Williams E medium (Sigma-Aldrich, Missouri, U.S.) supplemented with 20 mM HEPES, 2 mM glutamine, 10 nM insulin, 100 nM dexamethasone, 10 mM gentamicin, and 55 nM amphotericin B for 6 days at 37°C in 5% CO_2_, renewing and saving the medium every 24 h. In saved media, free glycine- or taurine-conjugated chenodeoxycholic, lithocholic, deoxycholic, cholic, and ursodeoxycholic acid were analyzed using liquid chromatography-tandem mass spectrometry as described previously^17^.

### Hepatocyte Transplantation into Immunodeficient Mice

Hepatocytes were transplanted into Fah^-/-^/Rag2^-/-^/Il2rg^-/-^/NOD (FRGN) mice to investigate engraftment and potential maturation of hepatic metabolism. FRGN mice are fumarylacetoacetate hydrolase (Fah) deficient, which advantageous for Fah-proficient donor cells. The mice are profoundly immunocompromised, with knockout of the recombination activating gene 2 (Rag 2) and the interleukin 2 receptor genes (IL2rg), allowing them to accept human cell transplant^18,19^. In addition, the FRGN mice were bred into a non-obese diabetic (NOD) background to enhance engraftment of donor cells.

**Figure 1.**
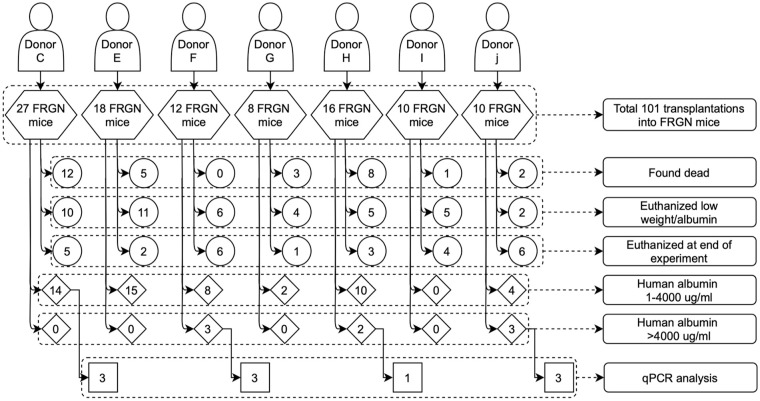
Flowchart describing number of FRGN (Fah^-/-^/Rag2^-/-^/Il2rg^-/-^/NOD) mice transplanted, amount of repopulation (1mg/mL = 20% repopulation), and the final number of mice used for qPCR (quantitative polymerase chain reaction) analysis. Fah: fumarylacetoacetate hydrolase; Rag 2: recombination activating gene 2; Il2rg: interleukin 2 receptor genes; NOD: non-obese diabetic.

Human hepatocyte xeno-transplantation into FRGN mice was performed as described previously^20^. Hepatocyte engraftment was assessed by monitoring human serum albumin using the human albumin ELISA Quantitation Kit (Bethyl Laboratories, Texas, U.S.) and considering that 1 mg/mL of human albumin correlates to approximately 20% repopulation^20^. Hepatic tissue from animals showing repopulation with no signs of suffering (i.e., losing weight) were used in the study. Euthanasia occurred on average 110 days post-cell injection after animals showed presence of serum human albumin. Murine “humanized” livers were extirpated and snap frozen in liquid nitrogen. The average albumin concentration was 3.2 ± 2.5 mg/mL, corresponding to approximately 64% repopulation (see Supplementary Information Fig. S1). A total of 101 FRGN mice were transplanted with hepatocytes from seven different donors, with a single donor per animal (Fig. 1).

### RNA Extraction and qPCR

Total RNA was extracted from snap-frozen donor liver tissue and humanized murine liver tissue using Trizol reagent (Invitrogen, Massachusetts, U.S.) following the manufacturer’s protocol. The cDNA was synthesized using the High Capacity Reverse Transcriptase Kit (Invitrogen, Massachusetts, U.S.) following the manufacturer’s protocol. Quantitative polymerase chain reaction (qPCR) analysis of genes of interest (see Supplementary Information Table 1) was performed in duplicate as described previously^20,21^. Human cyclophilin A (PPIA) mRNA was used for normalization. Human-specific TaqMan primers (Thermo Fisher Invitrogen, Massachusetts, U.S) were used to ensure that no cross-reaction occurred with mouse homologs. Samples were analyzed using mouse cDNA as a negative control.

Relative gene expression was analyzed using the comparative Ct method with 2^(-Ct)^, and PPIA as an internal control^22^.

### Statistical Analysis

Values are presented as medians with interquartile ranges (IQRs). Differences in viability or metabolic activity between diagnostic groups were analyzed by the Mann-Whitney test. Correlations between ischemia times and viability were assessed using Pearson’s correlation. To assess the significance in changes in the relative gene expression, two-sample T tests were used. For differences with non-normal distribution, the Mann-Whitney test was used. P<0.05 was considered significant. Data were analyzed using Prism software, version 8 (GraphPad Software Inc., California, U.S.).

### Ethical Approval

This study was approved by the Regional Ethical Review Boards in Stockholm (2012/1722-31/2 and 2019-00489). For transplantation of hepatocytes into a humanized mouse model, the ethical permit was approved by the local animal ethical committee in Stockholm (ID400 42-17).

## Results

**Figure 2.**
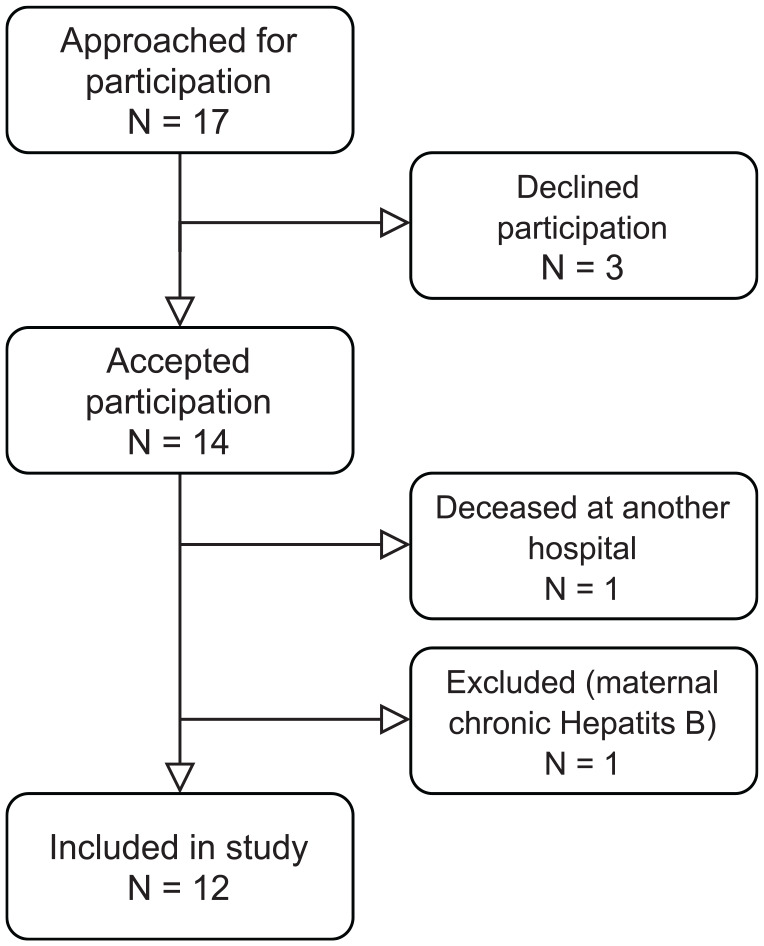
Flow chart describing the inclusion process of the 12 donors. After a prolonged agonal phase, one of the excluded patients was moved to another hospital, where the patient later deceased, thereby excluded from the study.

Seventeen families were approached for possible donation. Three families declined participation but 14 accepted, 2 of which were excluded. One patient was transferred to another hospital where they died after presenting with a prolonged agonal phase following withdrawal of life-sustaining treatment. One patient was excluded due to maternal chronic hepatitis B virus infection. Thus, 12 livers were procured (Fig. 2); the donors are referred to in the study as A-L. Cardiac valves were also procured from two donors (B and C) for clinical use.

### Donor Characteristics

Donors A, C, F, J, and K were delivered by emergency cesarean section. In donors B, E, and G, prenatal diagnosis had been carried out, and cesarean section was planned. The remaining four donors (D, H, I, and L) were delivered vaginally.

Causes of death were asphyxia (n = 7), organ failure following sepsis (n = 2), vena galena malformation (n = 1), anencephaly (n = 1), and Meckel Gruber’s syndrome (n = 1). Withdrawal of life-sustaining treatment comprised extubation (n = 7), inotropic and positive pressure ventilation treatment removal (n = 2), and cessation of resuscitation attempts (n = 1). In two patients with prenatal diagnosis (Meckel Gruber syndrome and anencephaly), life-sustaining treatment was never initiated after delivery. Two donors were part of multiple births with healthy twins (donors E and G). Four donors were female. Eight donors were born at term (>37 weeks gestation) and four were born preterm, between gestational age 25–36 weeks. Median gestational age was 37^+4^ (IQR: 35^+5^–38^+5^) weeks^+days^. Median age at donation was 5.5 (IQR: 2.5–8.5) days. Median body weight at donation was 2995 (IQR: 2126–3799) grams, with the livers weighing 98 (IQR: 63–120) grams, 1.9%–6.3% of the total body mass (Table 1).

**Table 1.** Donor Characteristics.

Donor #	Diagnosis	Weight at donation (grams)	Gestational age (w+d)	DoL (days)	Sex	Vasopressor treatment	Cardiac tissue
A	Asphyxia	2941	35+4	18	M	No	No
B	V. Galeni malformation	3982	37+1	7	M	Yes	Yes
C	Asphyxia	3800	38+1	5	M	No	Yes
D	Sepsis	3230	38+6	2	F	Yes	No
E	Anencephaly	1583	36+1	0	M	No	No
F	Asphyxia	3795	41+2	4	F	Yes	No
G	MGS	2264	37+3	0	M	No	No
H	Asphyxia	3049	37+5	6	F	No	No
I	Sepsis (NEC)	523	25+1	14	F	Yes	No
J	Asphyxia	4581	39+1	5	M	Yes	No
K	Asphyxia	2080	34+0	7	M	No	No
L	Asphyxia	2675	38+5	9	M	No	No

Table describing demographic characteristics of the 12 included donors. DoL: day of life; MGS: Meckel Gruber Syndrome; NEC: necrotizing enterocolitis.

### Hepatocyte Isolation, Biochemical Values, Viability, Yield, and Ischemia Times

Hepatocytes procured from donor D had a viability of 0%. Donor D died of *Escherichia coli* sepsis and no viable cells were obtained. Hepatocyte cultures showed growth of bacteria and the case was excluded from further analysis and results.

Median ALT was 0.55 (IQR: 0.49–1.63; ref <0.85 µkat/L). The INR was 1.5 (IQR: 1.2–1.7; ref <2). Total bilirubin was 54 (IQR: 26–149; ref <200 µmol/L;). Median agonal time was 50 (IQR: 10–79) minutes. Median WIT was 190 (IQR: 80–210) minutes, CIT 164 (IQR: 120–226) minutes, and total ischemic time 334 (IQR: 226–436) minutes. Median viability was 86% (IQR 71%–91%). The median yield was 6.9 (IQR: 3.4–12.8) x10^6^ hepatocytes/g of liver tissue (Table 2).

**Table 2.** Donor Laboratory Values, Recorded Time Points, as Well as Characteristics of Isolated Hepatocytes.

Donor #	ALT µkat/L	INR	Bilirubin µmol/L	Agonal time (min)	WIT (min)	CIT (min)	Liver weight (g)	Viability (%)	Yield (x10^6^/g)
A	0.56	1.3	30	79	382	672	55	38	0.1
B	0.13	1.7	103	10	210	226	111	91	10.7
C	0.54	N/A	69	60	83	251	80	79	23.7
D^a^	0.88	1.8	104	0	185	94	158	0	0
E	N/A	N/A	N/A	64	208	164	63	95	12.8
F	0.95	1.1	22	41	80	63	159	88	24
G	N/A	N/A	N/A	93	162	120	107	93	4.5
H	1.85	1.4	10	50	190	132	98	86	6.5
I	N/A	1.5	54	32	205	155	33	71	3.4
J	3.26	1.7	34	556	732	94	120	61	2.5
K	0.52	1.2	194	3	49	177	86	83	6.9
L	0.48	1.8	236	10	40	164	122	90	9.3

Local reference value for ALT <0.85, INR <2, Bilirubin <200. ALT: alanine amino transferase; INR: international normalized ratio; WIT: warm ischemia time; CIT: cold ischemia time.

a Not included in analysis due to culture growth.

**Figure 3.**
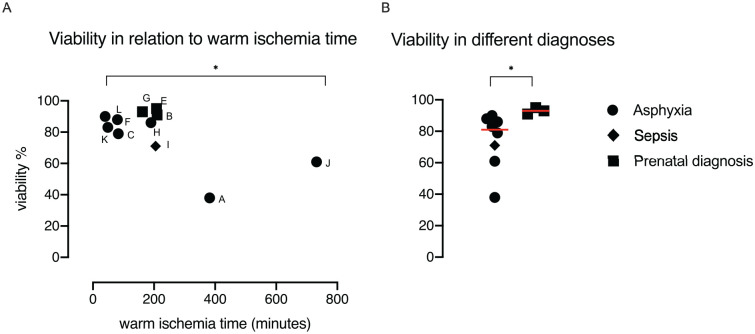
(A) Viability of neonatal hepatocytes (%) in relation to warm ischemia time (minutes). Correlation assessed using Pearson’s correlation. (B) Median viability of neonatal hepatocytes (%) grouped according to diagnose, line indicating median.

A correlation was observed between WIT and viability (R = -0.6, p = 0.04; Fig. 3). Furthermore, hepatocytes from donors prenatally diagnosed with conditions not compatible with life had higher viability than cases of asphyxia and sepsis (p = 0.01; Fig. 3). No difference in viability was found between term and preterm donors. Viability was 88% (IQR: 79%–91%) for term and 77% (IQR: 46%–92%) for preterm (p = 0.53).

**Figure 4.**
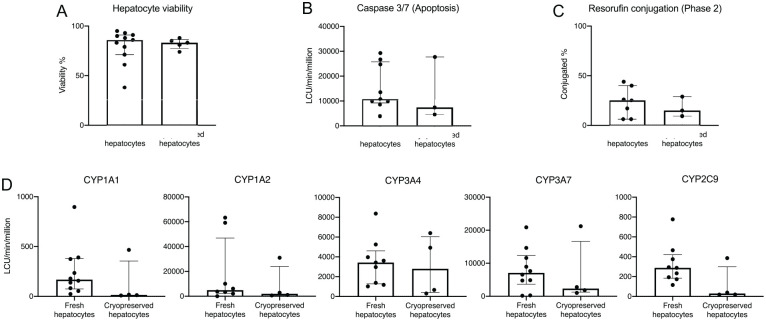
Comparisons between fresh and cryopreserved neonatal hepatocytes in (A) viability, (B) apoptosis (caspase 3/7), (C) phase 2 metabolism (resorufin conjugation) and (D) phase 1 metabolism (CYP 1A1, 1A2, 3A4, 3A7, 2C9) as measured in luminescent counting units per minute normalized to million viable cells (LCU/min/million), using Luciferin assays. Bars represent median value, error bars indicating interquartile range. LCU: luminescent counting units.

Neonatal hepatocytes maintained their viability following cryopreservation. The median viability following cryopreservation was 83% (IQR 81%-84%; selected cases: C, E, F, G, H). No significant difference in viability was observed between fresh and cryopreserved hepatocytes, with a loss of viability of 1–7% (Fig. 4A).

### Metabolic Activity

The median caspase-3/7 activity, which reflects the levels of apoptosis, was 10,716 (IQR: 9826–24,732) LCU/min/million for fresh cells and 7394 (IQR: 5925–17,556) LCU/min/million after cryopreservation (Fig. 4B).

Levels of phase 1 metabolism for the CYP1A family were 168 (IQR: 96–340) LCU/min/million in fresh cells and 16 (IQR: 11–129) LCU/min/million after cryopreservation for 1A1, and 4926 (IQR: 3044–22,322) LCU/min/million in fresh cells and 1961 (IQR: 941–9909) LCU/min/million after cryopreservation for 1A2 (Fig. 4D). There are two different isoforms in the CYP3A family: adult isoform CYP3A4 and fetal isoform CYP3A7. The median CYP3A4 activity was 3427 (IQR: 1378–3968) LCU/min/million in fresh cells and 2795 (IQR: 576–5294) LCU/min/million after cryopreservation. For CYP3A7, the median activity was 7091 (IQR: 4787–10,944) LCU/min/million in fresh cells and 2325 (IQR: 1652–7393) LCU/min/million after cryopreservation (Fig. 4D). For CYP2C9, an activity of 288 (IQR: 194–375) LCU/min/million was measured in fresh cells and 30 (IQR: 19–125) LCU/min/million in cryopreserved cells (Fig. 4D).

In phase 2 metabolism, hepatocytes presented a median conjugation of 25% (IQR: 11%–33%) in fresh cells. After cryopreservation, a conjugation of 15% (IQR: 8%–18%) was measured (Fig. 4C).

None of the differences in metabolic activity measured between fresh and cryopreserved cells were significant.

### Bile Acid Synthesis

**Figure 5.**
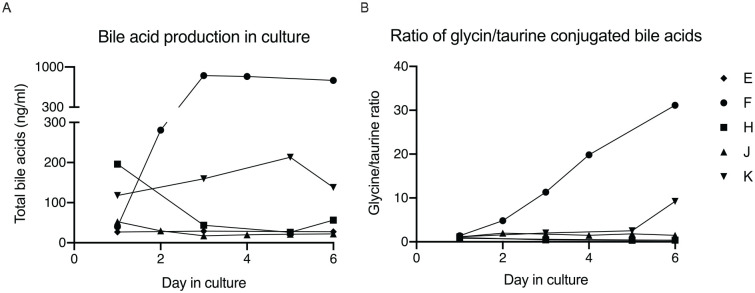
(A) Describing total bile acid production of neonatal hepatocytes in culture. (B) Describing ratio of glycine to taurine conjugated bile acids over time of cultured neonatal hepatocytes. Neonatal hepatocytes were plated on Matrigel, cultured for six days.

For five donors (E, F, H, J, and K), the hepatocytes were cultured, and the media collected. Neonatal hepatocytes produced and secreted bile acids in culture (Fig. 5). The ratio of glycine- to taurine-conjugated bile acids increased in culture. This was particularly noticeable with donors F and K, as bile acid production was markedly increased during the culture period.

### Hepatocyte Transplantation in FRGN Mice

**Figure 6.**
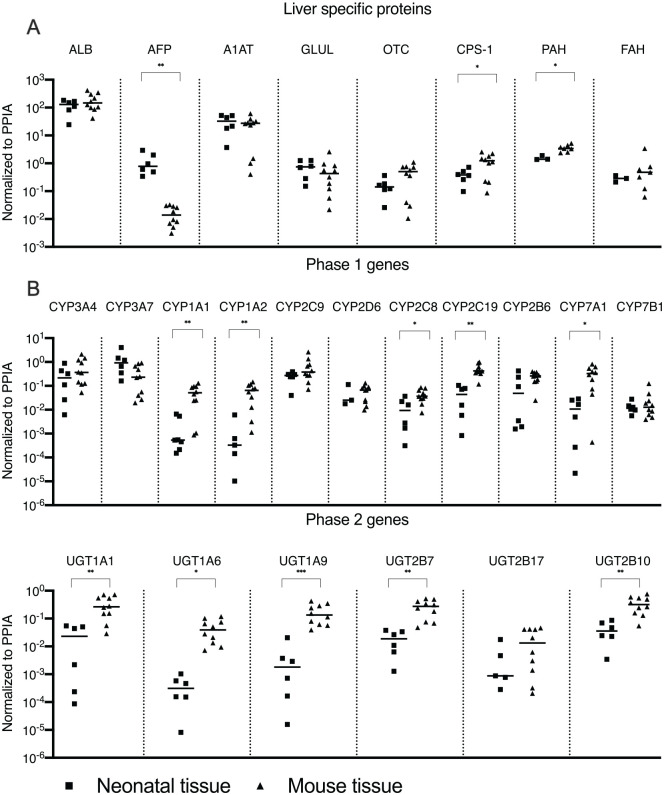
Y axis show relative gene expression normalized to endogenous control Human Cyclophilin A (PPIA). X axis is divided into respective gene showing relative expression of snap-frozen neonatal tissue (squares) and chimeric mouse tissue (triangle). Line indicating median. (A) Displaying relative expression of genes responsible for producing plasma proteins. (B) Displaying genes responsible for phase 1 metabolism. (C) Displaying genes responsible for phase 2 metabolism. ALB: albumin; AFP: alpha-fetoprotein; A1AT: alpha-1 antitrypsin; GLUL: Glutamate-ammonia ligase (glutamine synthetase); OTC: ornithine transcarbamylase; CPS-1: carbamoyl phosphate synthase 1; PAH: phenylalanine hydroxylase; FAH: fumarylacetoacetate hydrolase; UGT: UDP glucuronosyltransferase.

The expression of genes responsible for the production of albumin (ALB), A1AT, OTC, CPS-1, and phenylalanine hydroxylase (PAH) was maintained after transplantation into FRGN mice. An increase in CPS-1 and PAH gene expression was observed after transplantation. Hepatocytes presented high expression of alpha-fetoprotein (AFP) prior to transplantation, indicating immaturity. A significant decrease in AFP expression occurred in vivo after transplantation, exhibiting signs of continuous maturation following engraftment (Fig. 6A).

Post-transplantation, an increase was observed in the expression of CYP1A1 and CYP1A2, enzymes responsible for the metabolization of drugs and environmental chemicals. Of the two main isoforms in the CYP3A subfamily, CYP3A7 decreased and CYP3A4 increased after transplantation, but these changes were not significant. We observed an increase in the gene expression of CYP7A1, the rate limiting enzyme responsible for the synthesis of bile acids (Fig. 6B).

A significant increase in gene expression occurred for all UGT enzymes following transplantation, except for UGT2B17 (Fig. 6C). No significant differences were observed in genes encoding transcriptional factors. In genes encoding transport proteins, a significant increase in expression was observed for NTCP and a significant decrease for MRP4. In pluripotency-associated genes, a significant decrease in DLK-1 was observed (see Supplementary Information Fig. S2).

## Discussion

In this neonatal organ donation after circulatory death program to procure and evaluate hepatocytes for transplantation, hepatocytes isolated from neonatal donors were of sufficient quality, regardless of gestational age, as assessed by viability and function, fulfilling the release criteria for clinical transplantation at our center. The hepatocytes exhibited resilience to cryopreservation. Metabolic activity was evident in isolated neonatal hepatocytes, though the activity decreased after cryopreservation. Hepatocytes produced bile acids in culture, a highly specific marker of liver function. In addition, transplantation of hepatocytes into FRGN mice showed engraftment, functionality, and signs of maturation.

A positive experience was the high participation rate in our study. Previous reports suggested that hospital staff may be reluctant to enquire about organ donation because they assume unwillingness^23^. Our experience was the opposite, including cases in which the parents themselves requested organ donation. However, it is important to acknowledge that it was up to ward staff to identify and approach potential participants, and it is likely that selection bias was present.

Three factors contributed to the prolonged WITs observed in our study. Primarily we sought not to alter the already established clinical routine, but to explore what would be possible within the current framework. Hence bereaved parents could spend as much time as they wished with their child prior to the donation procedure. Secondly, patients were not continuously monitored; instead, examinations were done at intervals. Lastly, Death was declared according to the regulations of the Swedish National Board of Health and Welfare, which specifies an observational period of 20 minutes when determining death in newborns^12^.

Comparatively, Lee et al isolated hepatocytes from neonatal donors with an average viability of 89.4±1.8% and yield of 9.3±2.0 x10^6^ cells/g^7^. Tolosa et al isolated neonatal hepatocytes with a mean viability of 87±4% and yield of 21.2±14.4 x10^6^ cells/g^6^. Analyzing the raw data available from Lee et al and Tolosa et al, we found no significant difference in mean hepatocyte viability or yield compared to our results. Compared to Lee et al, our group had a significantly longer WIT (212±198 minutes vs. 35±30 minutes). However, Lee et al had a significantly longer CIT (334±148 minutes vs. 213±198 minutes), and no difference was seen in total ischemic time between the two studies. In Tolosa et al, the WIT was limited to 30 minutes (though not explicitly reported) and the CIT was shorter than in our study (113±33 minutes). Thus, a significantly shorter total ischemic time would have been present in Tolosa et al. In our study, despite the extended WIT, the hepatocytes still met the release criteria. Seven of the 12 cases had WIT ≥90 minutes; 6 of these cases had viability >60%. Viability of ≥50–60% has previously been described as the minimum for transplantation^2,24^. In contrast to formal DCD programs, our study shows that, in cases with extended WIT in which bereaved parents wish to donate their infant’s organs, hepatocyte donation can still be an option. However, if a neonatal donation program utilizing solid organs is to be implemented successfully, ischemia times have to be drastically reduced.

Although the yield per gram of liver tissue was high, a concern is the total yield, as neonatal livers are considerably smaller than adult livers. The median liver weight in our study was 98 g, compared to the weight of adult livers, which is approximately 1500 g. The total yield would amount to 676 x 10^6^ hepatocytes (98 x 6.9). In a clinical setting, 200 x10^6^ adult hepatocytes are needed per kilogram of body weight when transplanting hepatocytes^2^. On average, hepatocytes isolated from a neonatal donor would suffice for a recipient weighing up to 3.3 kg. However, a best-case scenario exemplified by donor F was 3822 x10^6^ hepatocytes, which would suffice for a recipient weighing up to 19 kg.

The highest viability was obtained from donors with a prenatal diagnosis. We speculate that this difference in viability is due to hypoxic damage to the liver in asphyxia and multiorgan failure following sepsis.

A theoretical advantage of hepatocyte transplantation compared to solid organ transplantation is the possibility of cryopreserving hepatocytes, making them available as on-demand treatment for patients suffering from acute liver failure. Engrafted hepatocytes can act as supportive treatment, bridging to transplantation or until the native liver can heal and resume function. This requires hepatocytes to have a high tolerance for cryopreservation. Our results indicate that neonatal hepatocytes tolerate cryopreservation to a great extent (Fig. 4A). Comparatively, our long-term experience with adult human hepatocytes indicates a frequent loss after cryopreservation of 15%–25%, with an average viability of 55% ± 15% (unpublished data). Neonatal hepatocytes demonstrated sufficient metabolic capacity compared to our previous measurements in adult cells^16^. However, the metabolic activity decreased after cryopreservation. Emphasizing the fact that viability does not necessarily reflect hepatocyte functionality, hence it is important to assess hepatocytes in both regards. Furthermore, neonatal hepatocytes were capable of producing bile acids in culture, a highly specific liver function.

To the best of our knowledge, this is the first study to evaluate neonatal hepatocytes in an animal model. The expression of genes responsible for the most common inborn metabolic diseases, A1AT, OTC, and CPS-1, was similar in neonatal liver tissue and previous reports for adult liver tissue^21^. In line with previously published results, neonatal hepatocytes presented signs of immaturity pre-transplantation^6^. Compared to previously published data for adult hepatocytes, neonatal hepatocytes expressed high levels of AFP and low levels of genes responsible for phase 1 and 2 metabolism^21^. Following transplantation, AFP expression significantly decreased and the expression of many of the genes responsible for phase 1 and 2 metabolism significantly increased (none decreased), almost reaching adult levels. These results suggest continual maturation of neonatal hepatocytes after transplantation into humanized mice.

In conclusion, livers from neonatal donors after prolonged WIT can provide excellent hepatocytes, even in infants born preterm. The hepatocytes demonstrate resilience to cryopreservation and expression of target genes responsible for causing metabolic monogenic diseases, even after transplantation. Although neonatal hepatocytes exhibit signs of immaturity, we found signs of maturation in vivo. Therefore, hepatocytes isolated from neonatal donors seem to be a good alternative source of hepatocytes for transplantation.

## Supplemental Material

sj-docx-1-cll-10.1177_09636897211069900 – Supplemental material for Procurement and Evaluation of Hepatocytes for Transplantation From Neonatal Donors After Circulatory Death Click here for additional data file. Supplemental material, sj-docx-1-cll-10.1177_09636897211069900 for Procurement and Evaluation of Hepatocytes for Transplantation From Neonatal Donors After Circulatory Death by Emil Bluhme, Ewa Henckel, Roberto Gramignoli, Therese Kjellin, Christina Hammarstedt, Greg Nowak, Ahmad Karadagi, Helene Johansson, Öystein Jynge, Maria Söderström, Björn Fischler, Stephen Strom, Ewa Ellis, Boubou Hallberg and Carl Jorns in Cell Transplantation
