# Supplementary material for: Procurement and Evaluation of Hepatocytes for Transplantation From Neonatal Donors After Circulatory Death
Source: Cell Transplant. 2022 Jan 30;31:09636897211069900. doi: 10.1177/09636897211069900 (PMC8811420; doi:10.1177/09636897211069900)
Supplement: sj-docx-1-cll-10.1177_09636897211069900 – Supplemental material for Procurement and Evaluation of Hepatocytes for Transplantation From Neonatal Donors After Circulatory Death [file sj-docx-1-cll-10.1177_09636897211069900.docx]

**Supplementary information**

**Supplementary Table 1.** TaqMan assays used for qPCR (quantitative polymerase chain reaction) analyses. Gene abbreviation and gene name provided along with assay ID.

|  | **Gene abbreviation** | **Gene name** | **Assay ID** |
| --- | --- | --- | --- |
|  | PPIA | Cyclophilin A (peptidylprolyl isomerase A) | Hs99999904_m1 |
| Liver specific plasma proteins/metabolic genes | ALB | Human albumin | Hs00609411_m1 |
|  | AFP | Alpha-fetoprotein | Hs00173490_m1 |
|  | A1AT | Alpha-1 antitrypsin (SERPINA1) | Hs01097800_m1 |
|  | GLUL | Glutamate-ammonia ligase (glutamine synthetase) | Hs01018343_g1 |
|  | OTC | Ornithine carbamoyltransferase | Hs00166892_m1 |
|  | CPS-1 | Carbamoyl-phosphate synthetase 1 | Hs00157048_m1 |
|  | PAH | Phenylalanine hydroxylase | Hs00609359_m1 |
|  | FAH | Fumarylacetoacetate hydrolase | Hs00908445_m1 |
|  |  |  |  |
| Phase 1 genes | CYP1A1 | Cytochrome P450 family 1 subfamily A member 1 | Hs00153120_m1 |
|  | CYP1A2 | Cytochrome P450 family 1 subfamily A member 2 | Hs01070374_m1 |
|  | CYP2B6 | Cytochrome P450 family 2 subfamily B member 6 | Hs03044634_m1 |
|  | CYP2C8 | Cytochrome P450 family 2 subfamily C member 8 | Hs00258314_m1 |
|  | CYP2C19 | Cytochrome P450 family 2 subfamily C member 19 | Hs00426380_m1 |
|  | CYP2D6 | Cytochrome P450 family 2 subfamily D member 6 | Hs02576167_m1 |
|  | CYP3A4 | Cytochrome P450 family 3 subfamily A member 4 | Hs00430021_m1 |
|  | CYP3A7 | Cytochrome P450 family 3 subfamily A member 7 | Hs00426361_m1 |
|  | CYP7A1 | Cytochrome P450 family 7 subfamily A member 1 | Hs00167982_m1 |
|  | CYP7B1 | Cytochrome P450 family 7 subfamily B member 1 | Hs00191385_m1 |
|  |  |  |  |
| Phase 2 genes | UGT1A1 | UDP glucuronosyltransferase 1 family member A1 | Hs02511055_s1 |
|  | UGT1A6 | UDP glucuronosyltransferase 1 family member A6 | Hs01592477_m1 |
|  | UGT1A9 | UDP glucuronosyltransferase 1 family member A9 | Hs02516855_sH |
|  | UGT2B7 | UDP glucuronosyltransferase 2 family member B7 | Hs00426592_m1 |
|  | UGT2B10 | UDP glucuronosyltransferase 2 family member B10 | Hs02556282_s1 |
|  | UGT2B17 | UDP glucuronosyltransferase 2 family member B17 | Hs00854486_sH |
|  |  |  |  |
| Transcription factors | LXRa | Liver X receptor alpha (NR1H3) | Hs00172885_m1 |
|  | LXRb | Liver X receptor beta (NR1H2) | Hs01027215_g1 |
|  | FXR | Farnesoid X nuclear receptor (NR1H4) | Hs00231968_m1 |
|  | PXR | Pregnane X nuclear receptor (NR1I2) | Hs01114267_m1 |
|  | CAR | Constitutive androstane nuclear receptor (NR1I3) | Hs00231959_m1 |
|  | PPARa | Peroxisome proliferator-activated receptor alpha (NR1C1) | Hs00947536_m1 |
|  | PPARg | Peroxisome proliferator-activated receptor gamma (NR1C3) | Hs01115513_m1 |
|  | HIF1A | Hypoxia-inducible factor 1-alpha | Hs00153153_m1 |
|  | HNF1a | Hepatic nuclear factor 1 alpha | Hs00167041_m1 |
|  | HNF1b | Hepatic nuclear factor 1 beta | Hs00172123_m1 |
|  | HNF3a | Hepatic nuclear factor 3 alpha (FOXA1) | Hs04187555_m1 |
|  | HNF3b | Hepatic nuclear factor 3 beta (FOXA2) | Hs00232764_m1 |
|  | HNF4a | Hepatic nuclear factor 4 alpha | Hs00230853_m1 |
|  | HNF6 | Hepatic nuclear factor 6 (ONECUT1) | Hs00413554_m1 |
|  | AHR | Aryl hydrocarbon receptor | Hs00169233_m1 |
|  | GR | Glucocorticoid receptor (NR3C1) | Hs00353740_m1 |
|  |  |  |  |
| Transporters | NTCP | Sodium/bile acid cotransporter 1 (SLC10A1) | Hs00914889_m1 |
|  | P-GP | ATP-binding cassette, B1 (ABCB1 - MDR1) | Hs00184500_m1 |
|  | MRP2 | ATP-binding cassette, C2 (ABCC2) | Hs00166123_m1 |
|  | MRP3 | ATP-binding cassette, C3 (ABCC3) | Hs00978473_m1 |
|  | MRP4 | ATP-binding cassette, C4 (ABCC4) | Hs00988717_m1 |
|  | BSEP | ATP-binding cassette, B11 (ABCB11) | Hs00184824_m1 |
|  | BCRP | ATP-binding cassette, G2 (ABCG2) | Hs00184979_m1 |
|  |  |  |  |
| Pluripotency genes | DLK1 | Delta-like 1 homolog | Hs00171584_m1 |
|  | NANOG | Nanog homeobox | Hs04260366_g1 |
|  | SOX2 | SRY (sex determining region Y)-box 2 | Hs01053049_s1 |
|  | SOX9 | SRY (sex determining region Y)-box 9 | Hs01001343_g1 |
|  | SOX17 | SRY (sex determining region Y)-box 17 | Hs00751752_s1 |
|  | OCT4 | Octamer-binding transcription factor | Hs00742896_s1 |

|  |
| --- |
| **Figure S1.** Showing serum human albumin obtained from FRGN mice used in the study. Y axis showing serum human albumin concentration in ng/ml. X axis showing days from transplantation. |

**Figure S2.** Y axis show relative gene expression normalized to endogenous control Human Cyclophilin A (PPIA). X axis is divided into respective gene showing relative expression of snap-frozen neonatal tissue (squares) and chimeric mouse tissue (triangle). Line indicating median
